# Role of [^18^F]FDG PET/CT in the management of follicular cell-derived thyroid carcinoma

**DOI:** 10.1186/s40644-024-00791-8

**Published:** 2024-10-28

**Authors:** Klaudia Zajkowska, Paulina Cegla, Marek Dedecjus

**Affiliations:** Department of Endocrine Oncology and Nuclear Medicine, Maria Skłodowska-Curie National Research Institute of Oncology, Roentgena Street 5, Warsaw, 02-781 Poland

**Keywords:** PET/CT, [^18^F]FDG, Thyroid cancer, Dedifferentiation, Thyroid incidentaloma, TENIS syndrome

## Abstract

Follicular cell-derived thyroid carcinomas constitute the majority of thyroid malignancies. This heterogeneous group of tumours includes well differentiated, poorly differentiated, and undifferentiated forms, which have distinct pathological features, clinical behaviour, and prognosis. Positron emission tomography with 2-[^18^F]fluoro-2-deoxy-D-glucose combined with computed tomography ([^18^F]FDG PET/CT) is an imaging modality used in routine clinical practice for oncological patients. [^18^F]FDG PET/CT has emerged as a valuable tool for identifying patients at high risk of poor clinical outcomes and for facilitating individualized clinical decision-making. The aim of this comprehensive review is to summarize current knowledge regarding the role of [^18^F]FDG PET/CT in primary diagnosis, treatment, and follow-up of follicular cell-derived thyroid carcinomas considering the degree of differentiation. Controversial issues, including significance of accidentally detected [^18^F]FDG uptake in the thyroid, the role of [^18^F]FDG PET/CT in the early assessment of response to molecular targeted therapies, and its prognostic value are discussed in detail.

## Background

Follicular cell-derived malignant neoplasms constitute the majority of thyroid malignancies. In the latest 5th edition of the World Health Organization (WHO) Classification of Thyroid Neoplasms, these tumours are classified according to pathological features, molecular background, and biological behaviour [1]. Follicular thyroid carcinoma (FTC) and invasive encapsulated follicular variant of papillary thyroid carcinoma (IEFVPTC) are characterized by a high incidence of *RAS*-like molecular alterations, whereas papillary thyroid carcinoma (PTC) and its numerous subtypes belong to the *BRAF*-like family of malignancies. Oncocytic carcinoma of the thyroid (OCA) (formerly Hürthle cell carcinoma) is considered a distinct entity because it has unique clinicopathologic and molecular characteristics. FTC, IEFVPTC, PTC, and OCA are traditionally referred to as well-differentiated thyroid carcinomas (WDTC), and are distinguished from other less differentiated types. In the latest edition of the WHO classification, a new category of high-grade non-anaplastic follicular-derived carcinomas was introduced for the first time; it includes poorly differentiated thyroid carcinoma (PDTC) and differentiated high-grade thyroid carcinoma (high-grade PTC, high-grade FTC, and high-grade OCA), which are characterized by the presence of tumour necrosis and/or increased mitotic activity, with an intermediate prognosis between well- and undifferentiated (anaplastic) carcinomas. Anaplastic thyroid carcinoma (ATC) remains the most dedifferentiated form of thyroid cancer. It is characterized by an extremely aggressive biological behaviour and poor prognosis [1].

Iodine-131 whole-body scan ([^131^I]I WBS) plays a pivotal role in the management of patients with WDTC. It is used in the postoperative evaluation of residual thyroid tissue, the detection of distant metastases, the determination of eligibility for radioactive iodine (RAI) therapy, and the assessment of treatment response [2]. During the process of dedifferentiation, thyroid cancer cells lose the capacity for RAI uptake and organification, which significantly limits the possibilities of using this radioisotope not only in diagnostics, but also for treatment, making the management of such patients challenging.

The introduction of positron emission tomography combined with multi-row computed tomography (PET/CT) into clinical practice has significantly changed the management of oncological patients. This scan allows simultaneous assessment of the patient’s organ anatomy and the location of pathological radiotracer uptake. Among several PET radiotracers with different targets and metabolic pathways, 2-[^18^F]fluoro-2-deoxy-D-glucose ([^18^F]FDG) is the best-characterized and most widely used. The value of [^18^F]FDG PET/CT has been confirmed in numerous studies, including in patients with follicular cell-derived thyroid carcinoma, particularly in poor- and undifferentiated types [3–6].

**Fig. 1:**
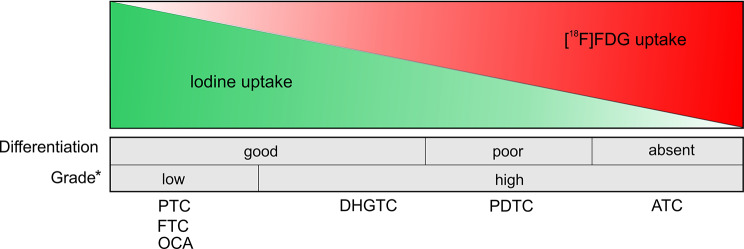
The “flip-flop” phenomenon of radioactive iodine and [^18^F]FDG uptake in follicular cell-derived thyroid carcinoma. Well-differentiated thyroid carcinomas with no high-grade features show high radioactive iodine uptake and low [^18^F]FDG uptake. By contrast, dedifferentiated (anaplastic) thyroid carcinomas are characterized by high [^18^F]FDG uptake and lack of radioactive iodine uptake. Differentiated high-grade thyroid carcinomas and poorly differentiated thyroid carcinomas may exhibit heterogenous uptake of both radiotracers. *mitotic activity, tumour necrosis

Dedifferentiation of thyroid cancer cells is a multi-step process that involves “early” and “late” genetic alterations, as well as epigenetic modifications [7]. This process entails a decrease or loss of sodium iodide symporter (NIS) expression and the upregulation of glucose transporter 1 expression [8]. This phenomenon results in an inverse relationship between RAI and [^18^F]FDG uptake (“flip-flop phenomenon”) in patients with thyroid cancer [9]. Most WDTCs are slow-growing tumours that can show negative [^18^F]FDG uptake [10]. The exception is OCA, which, more frequently than other WDTCs, does not take up RAI but shows positive [^18^F]FDG PET/CT results [11]. Next in the WHO classification, high-grade non-anaplastic thyroid carcinomas develop resistance to RAI and exhibit increased avidity in [^18^F]FDG PET in approximately 50% of cases [12, 13]. Finally, ATC consistently demonstrates high [^18^F]FDG uptake [14]. A graphical representation of the relationship between RAI and [^18^F]FDG uptake in follicular cell-derived thyroid carcinomas according to the degree of differentiation and the presence of high-grade features is shown in Fig. 1.

The aim of this comprehensive review is to summarize the most recent evidence on the role of [^18^F]FDG PET/CT in the primary diagnosis, treatment, and follow-up of patients with follicular cell-derived thyroid carcinoma.

### [^18^F]FDG-avid thyroid incidentaloma

The widespread use of [^18^F]FDG PET/CT has increased the frequency of incidental diagnosis of [^18^F]FDG-avid thyroid nodules, so-called thyroid incidentaloma. The incidental detection of radiotracer uptake within the thyroid gland during [^18^F]FDG PET/CT performed for purposes other than thyroid evaluation occurs in 1.9–4.3% of [^18^F]FDG PET/CT scans [15–19]. Radiotracer uptake in the thyroid gland can occur in a diffuse or focal pattern. Diffuse [^18^F]FDG uptake is associated with chronic thyroiditis, hypothyroidism, and Graves’ disease; it can also occur in patients without thyroid disease (considered a normal variant) [20, 21], where it is an indication for evaluation of thyroid function and anti-thyroid antibody levels, whereas fine needle aspiration biopsy (FNAB) is not required [2, 20].

By contrast, focal [^18^F]FDG uptake within the thyroid gland raises suspicion of malignancy, especially in lesions with a high standardized uptake value (SUV) [20]. In a recent meta-analysis by De Leijer et al. that included 50 studies and 640,616 patients, the pooled prevalence of focal [^18^F]FDG-avid thyroid incidentaloma was 2.22%, and the cumulative risk of malignancy was 30.8% [22]. Among malignant focal thyroid incidentalomas, the most common is PTC (82.6%), whereas other types including FTC, medullary thyroid carcinoma, ATC, lymphoma, and metastases to the thyroid account for 7%, 2.2%, 0.7%, 0.8% and 6.1%, respectively [22]. Approximately 21–26% of focal [^18^F]FDG PET thyroid incidentalomas subjected to FNAB yield nondiagnostic or indeterminate cytology results (Bethesda categories I, III, or IV) [22, 23].

The guidelines for the management of incidentally detected focal [^18^F]FDG-avid thyroid nodules are inconsistent. The 2015 American Thyroid Association (ATA) guidelines recommend the use of ultrasonography to confirm the presence of a nodule and FNAB for nodules ≥ 1 cm in size [2]. The British and Polish guidelines recommend FNAB for all nodules showing increased [^18^F]FDG uptake on PET regardless of size, although consideration of the clinical context is suggested [24, 25]. The recently published European Thyroid Association (ETA) guidelines recommend evaluating all incidentally detected PET-avid nodules according to standard thyroid nodule management principles, and making decisions regarding FNAB based on this evaluation [26].

An interesting voice in the discussion on thyroid incidentaloma is the consensus statement of a multidisciplinary group of experts led by Jonathan Wadsley on the management of incidentally discovered [^18^F]FDG-avid thyroid nodules in patients being investigated for other cancers [27]. The authors recommended that if a patient is unlikely to survive 5 years as a result of their age, long-term prognosis or co‐morbidities, further investigation is unlikely to be warranted. Exceptions include suspected metastasis to the thyroid that could affect the treatment of the known cancer, a thyroid incidentaloma larger than 4 cm, and concern that local progression of an undiagnosed thyroid malignancy may cause significant morbidity [27]. A less invasive approach seems reasonable given that the majority of PET-avid thyroid malignancies are PTCs, which are characterized by slow growth and a good prognosis [22]. Furthermore, the risk of thyroid cancer-related death in patients undergoing evaluation for another malignancy is extremely low [28].

### [^18^F]FDG PET/CT in the management of thyroid nodules with indeterminate cytology

The gold standard for the diagnosis of thyroid nodules is ultrasound examination, and in selected cases, FNAB. PET imaging, as well as CT or magnetic resonance imaging (MRI), are not recommended during the initial evaluation [2, 25, 26]. The primary limitation of FNAB is the occurrence of indeterminate results such as atypia of undetermined significance (Bethesda III category) and follicular neoplasm (Bethesda IV category) [29], which collectively account for 14–20% of all FNAB results [30, 31]. In such cases, repeat biopsy (only for Bethesda III), molecular diagnostics, or diagnostic surgery is recommended [29]. The estimated risk of malignancy is 22% for atypia of undetermined significance and 30% for follicular neoplasm [29], suggesting that many patients are overtreated and undergo unnecessary surgical treatment for benign lesions. Increasing evidence suggests that [^18^F]FDG PET/CT may be a cost-effective and clinically efficient alternative to conventional approaches [32–37].

The prospective, multicentre, randomized, controlled clinical trial *EfFECTS* showed that in patients with Bethesda III and IV thyroid nodules, [^18^F]FDG PET/CT is a highly accurate method to rule-out malignancy, with a sensitivity of 94% and a negative predictive value (NPV) of 95%. However, it is not suitable as a rule-in test [specificity, 40%; positive predictive value (PPV), 35%]. [^18^F]FDG PET/CT-based management (surveillance for [^18^F]FDG-negative nodules and diagnostic surgery for [^18^F]FDG-positive nodules) prevented 40% of futile diagnostic surgeries for benign nodules and 48% of surgeries in patients with non-oncocytic thyroid nodules [32]. In patients with oncocytic thyroid nodules, PET is not beneficial because nearly all nodules are [^18^F]FDG-positive [32, 38]. The conclusions drawn from the *EfFECTS* study are consistent with previous research demonstrating the high NPV of [^18^F]FDG PET/CT in the evaluation of nodules with indeterminate cytology results [33–35]. A recently published study by the same authors comparing the performance of [^18^F]FDG PET/CT with that of molecular diagnostics in Bethesda III and IV thyroid nodules indicated that the two methods are equally accurate for ruling out malignancy; however, their concordance was only 63% [39]. Finally, PET-based treatment is cost-effective compared with standard approaches, at least in the European setting, where the availability of multi-gene molecular tests is limited [36, 37].

In the preoperative evaluation of thyroid nodules with indeterminate cytology results, other radiopharmaceuticals, including [^18^F]fluorocholine ([^18^F]FCH) and [^99m^Tc]Tc-methoxyisobutylisonitrile ([^99m^Tc]Tc-MIBI), may also be useful. Studies have demonstrated that both [^18^F]FCH PET/CT and [^99m^Tc]Tc-MIBI scintigraphy exhibit high sensitivity and NPV, but poor specificity and PPV for predicting malignancy in indeterminate thyroid nodules [40–43]. The primary limitation of [^18^F]FCH is its high cost and reduced worldwide availability [44]. Conversely, [^99m^Tc]Tc-MIBI scintigraphy is a readily available and cost-effective procedure, but it is characterized by lower resolution and observer-dependent interpretation of results [41]. Interestingly, similar to [^18^F]FDG, both [^18^F]FCH and [^99m^Tc]Tc-MIBI, despite their differing uptake mechanisms, exhibit lower diagnostic performance in oncocytic thyroid nodules [40, 41].

### [^18^F]FDG PET/CT for preoperative evaluation

Preoperative evaluation of differentiated thyroid carcinoma (DTC) consists primarily of a thorough ultrasound examination of the neck with assessment of the thyroid gland and lymph nodes of the central and lateral neck compartments [2]. Ultrasound-detected suspicious lymph nodes should undergo FNAB prior to surgical treatment, and measurement of thyroglobulin (Tg) in the FNAB washout may also be helpful. Cross-sectional imaging scans (CT, MRI) should be performed in patients with locally advanced disease, e.g., in cases of vocal cord paresis; suspected infiltration of the trachea, oesophagus or major cervical vessels; and in patients with a large, rapidly growing tumour and suspected mediastinal lymphadenopathy, which may significantly affect the surgical treatment plan [2, 45]. [^18^F]FDG PET/CT is not recommended for routine preoperative evaluation of DTC [2, 25].

A meta-analysis by Kim et al. [46] showed that [^18^F]FDG PET/CT has low sensitivity (30%) despite its high specificity (94%) in detecting metastases of DTC to the lymph nodes of the neck. Another meta-analysis showed that [^18^F]FDG PET/CT has lower PPV, NPV and accuracy for cervical lymph node metastasis compared with the inexpensive and non-invasive neck ultrasound scan [47]. To the best of the authors’ knowledge, there are no reports on the usefulness of [^18^F]FDG PET/CT for the assessment of locoregional invasion of DTC. However, it is likely that PET will be inferior to contrast-enhanced CT and MRI, as indicated in studies of other head and neck cancers [48, 49]. [^18^F]FDG PET/CT is a highly sensitive and specific modality for detecting distant metastases of DTC [50, 51]. Nonetheless, when performed during preoperative assessment, it has a limited impact on clinical management. Patients with distant metastases detected during initial evaluation still undergo total thyroidectomy to facilitate subsequent RAI treatment [52]. This approach differs from that in most other malignancies, in which surgical treatment of the primary tumour in patients with metastatic disease is generally not recommended.

**Fig. 2:**
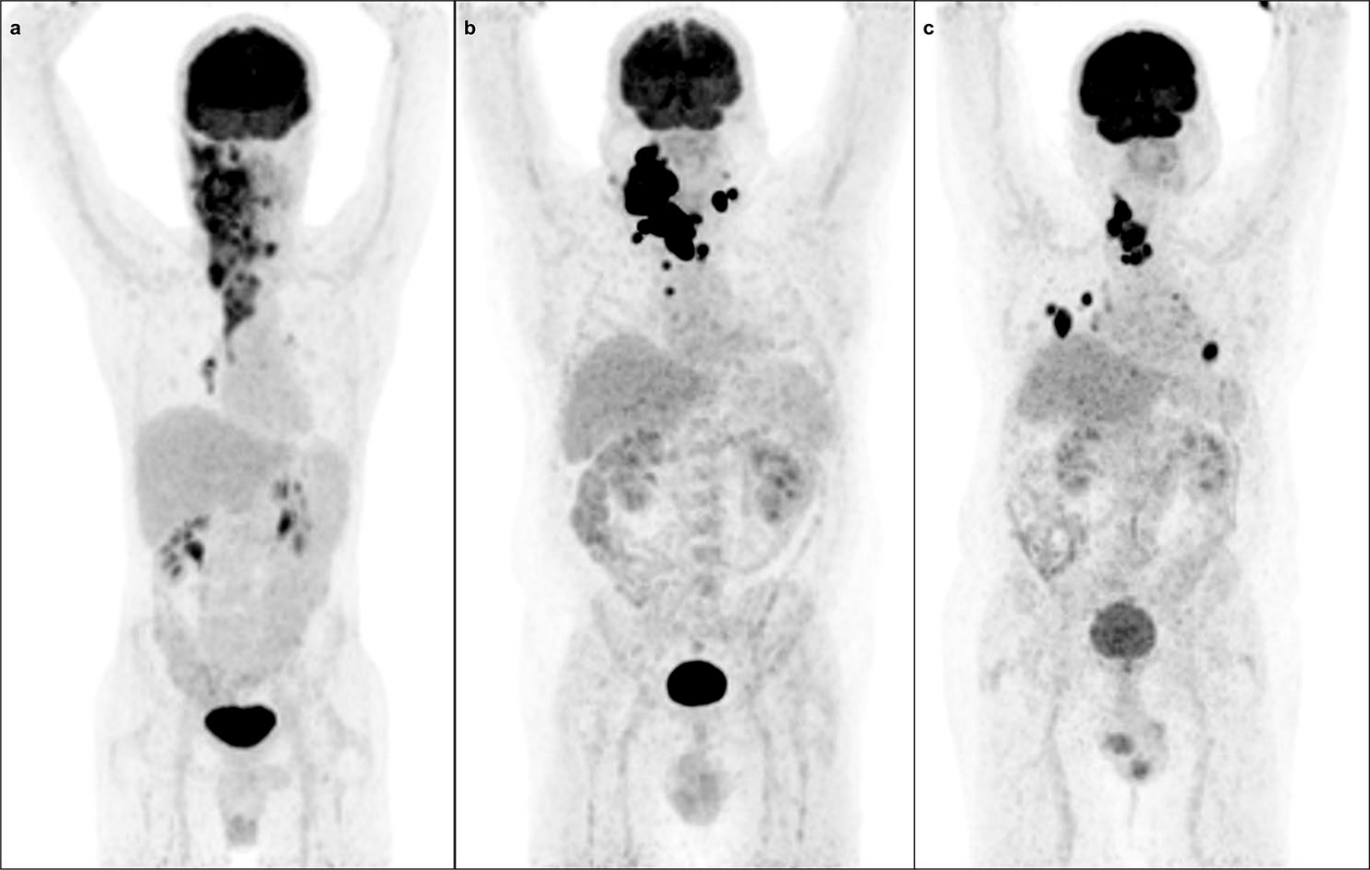
[^18^F]FDG PET/CT performed during initial evaluation. (**a**) A 34-year-old man with anaplastic thyroid carcinoma with metastases to the lymph nodes in the right lung hilum. (**b**) A 56-year-old man with squamous cell carcinoma of the thyroid (classified as anaplastic thyroid carcinoma according to the new WHO classification) with metastases to the mediastinal lymph nodes. (**c**) A 74-year-old man with anaplastic thyroid carcinoma with metastases to the lungs, mediastinal lymph nodes and lung hilum

In contrast to DTC, in ATC, [^18^F]FDG PET/CT plays a crucial role in determining the extent of disease, as well as in the surgical decision-making process. According to ATA guidelines, [^18^F]FDG PET/CT is the preferred imaging modality for the initial assessment of patients with ATC [53]. [^18^F]FDG PET/CT is more sensitive than contrast-enhanced CT in the detection of metastatic lymph nodes in the neck (100% vs. 57%) and mediastinum (100% vs. 56%), and metastasis to the lungs (100% vs. 83%), and it is more sensitive than bone scintigraphy for detecting bone metastases [14]. These findings were confirmed by Kim et al. [54], who performed [^18^F]FDG PET/CT on 40 patients with ATC for pre-treatment evaluation. Distant metastases were detected exclusively by [^18^F]FDG PET/CT in 15 patients (37.5%), of which 7 had lung metastases, 6 had bone metastases, 5 had them in extra-regional lymph nodes, and 1 in the brain [54]. However, [^18^F]FDG PET/CT is considered inferior to contrast-enhanced MRI for detecting brain metastases. Therefore, patients presenting with clinical symptoms suggesting the presence of brain metastases should undergo contrast-enhanced MRI as part of the initial staging [53]. Additionally, [^18^F]FDG PET/CT performed without intravenous contrast is insufficient for accurately assessing the extent of local invasion into the trachea, oesophagus, and great vessels, which is essential for evaluating the feasibility of surgical treatment. In patients with only locoregional disease (stage IVA/IVB), decisions regarding tumour resection should depend on the possibility of achieving satisfactory resection (R0/R1) and consider the patient’s quality of life and preferences. Extensive resections (including laryngectomy, tracheal resections, oesophageal resections, and major vascular or mediastinal resections) are generally not recommended because of the poor prognosis of ATC [53]. The results of [^18^F]FDG PET/CT have a direct impact on clinical management in 25–50% of patients with ATC [14, 55]. Examples of [^18^F]FDG PET/CT performed during the initial evaluation of patients with ATC are presented in Fig. 2.

### [^18^F]FDG PET/CT for postoperative staging and risk stratification

Postoperative staging of DTC is performed according to the latest edition of the Tumour-Node-Metastasis (TNM) Classification by the American Joint Committee on Cancer/Union for International Cancer Control (AJCC/UICC), which provides the most accurate estimation of disease-dependent survival [56]. In most centres that treat thyroid cancer, the risk of a poor clinical outcome is assessed using the classification proposed by the ATA, which incorporates data from the histopathological report (e.g., extent of tumour resection, histological subtype, presence of vascular invasion and extrathyroidal extension, and size of lymph node metastases), as well as the first clinical tests conducted after surgery and RAI treatment, if applicable [2]. The combined assessment of postoperative Tg levels and the result of the first WBS performed after the administration of the diagnostic or therapeutic dose of [^131^I]I enables early detection of metastases in 86.2% of patients [57]. However, expecially in patients with high-grade tumours, post-therapeutic scintigraphy can provide false-negative results and may not reveal the actual extent of the disease. In these cases, [^18^F]FDG PET/CT performed during RAI ablation therapy provides a more accurate assessment of disease extent, which facilitates changes in the treatment strategy and adequate monitoring of patients [58–62].

The largest published study evaluating the clinical utility of [^18^F]FDG PET/CT in the postoperative assessment of DTC is a retrospective study by Lee et al. that included 286 patients classified as intermediate or high risk according to ATA criteria [58]. All patients had [^18^F]FDG PET/CT scan performed concurrently with RAI treatment. In 39 (14%) patients, the [^18^F]FDG PET/CT scan detected additional recurrent or metastatic lesions that were not detected in the post-therapeutic [^131^I]I WBS, leading to a change in the treatment plan in 30 patients (10%). Both tumour stage and size were predictive of a positive [^18^F]FDG PET/CT result, and the highest frequency of additional PET-avid lesions was observed in patients with recurrent DTC (46%) and in patients with stage T3–T4N1 and tumour size > 2.0 cm (25%) [58].

Another study evaluated 38 patients with aggressive histological subtypes of DTC and PDTC without known persistent disease or distant metastases who had [^18^F]FDG PET/CT performed during postoperative RAI ablation [59]. Of 86 lesions detected in 20 (53%) patients, 41% were visible in [^18^F]FDG PET/CT only, 31% in the post-therapeutic [^131^I]I WBS only, and 28% in both modalities. The only statistically significant risk factor for a positive [^18^F]FDG PET/CT result was a stimulated Tg concentration measured at ablation > 10 ng/ml [59]. Other studies have also confirmed the clinical benefit of [^18^F]FDG PET/CT performed during ablative RAI therapy in high-risk patients, with suspected metastases or high postoperative Tg levels; this has led to a change in clinical management in 20–36% of patients [4, 60–64].

### [^18^F]FDG PET/CT during follow-up after initial treatment

The follow-up of patients after initial treatment (surgery +/- RAI treatment) of DTC aims to identify individuals with local recurrence or distant metastases; it consists of neck ultrasound examination, measurement of Tg levels together with anti-Tg antibodies (TgAb) and, in selected cases, diagnostic [^131^I]I WBS and other cross-sectional imaging studies [2]. The risk of thyroid cancer recurrence is estimated at 4.3% for PTC, 13.6% for FTC, and up to 39% for PDTC [65–67]. Furthermore, the risk of recurrence depends on tumour stage: patients in early stages (stages I and II) have significantly lower recurrence rates than those in advanced stages (stages III and IV) according to the AJCC/UICC TNM classification (7.2 vs. 28.2%, *p* < 0.05) [68]. Most recurrences occur within the first 5 years of follow-up, although recurrences after several decades of surgical treatment are also possible [69, 70].

As recommended by scientific societies, patients presenting with elevated serum Tg levels after effective initial treatment of DTC should undergo a neck ultrasound and diagnostic [^131^I]I WBS first [2, 71]. In most patients, this approach is sufficient. However, approximately 10% of patients exhibit elevated Tg levels without detectable lesions on the [^131^I]I WBS, a clinical situation referred to as thyroglobulin elevated negative iodine scintigraphy (TENIS) syndrome, which is an indication for [^18^F]FDG PET/CT [72]. According to meta-analysis, [^18^F]FDG PET/CT has the pooled sensitivity and specificity of 87% and 89%, respectively [51], in the detection of recurrence in patients with TENIS syndrome and is superior to conventional imaging studies [50]. [^18^F]FDG PET/CT performed in this context leads to changes in clinical management in 42–79% of patients, which most often consist of the cancellation of the next cycle of RAI therapy and a referral to surgical treatment, radiotherapy, or initiation of molecular targeted therapy [73–75].

Most scientific societies, including ATA, recommend the [^18^F]FDG PET/CT scan in DTC patients with serum Tg > 10 ng/ml [2, 25, 76]. However, the usefulness of [^18^F]FDG PET/CT in patients with lower Tg levels has been suggested [77, 78]. The National Comprehensive Cancer Network guidelines suggest considering [^18^F]FDG PET/CT when the stimulated Tg level is greater than 2–5 ng/ml [71].

**Fig. 3:**
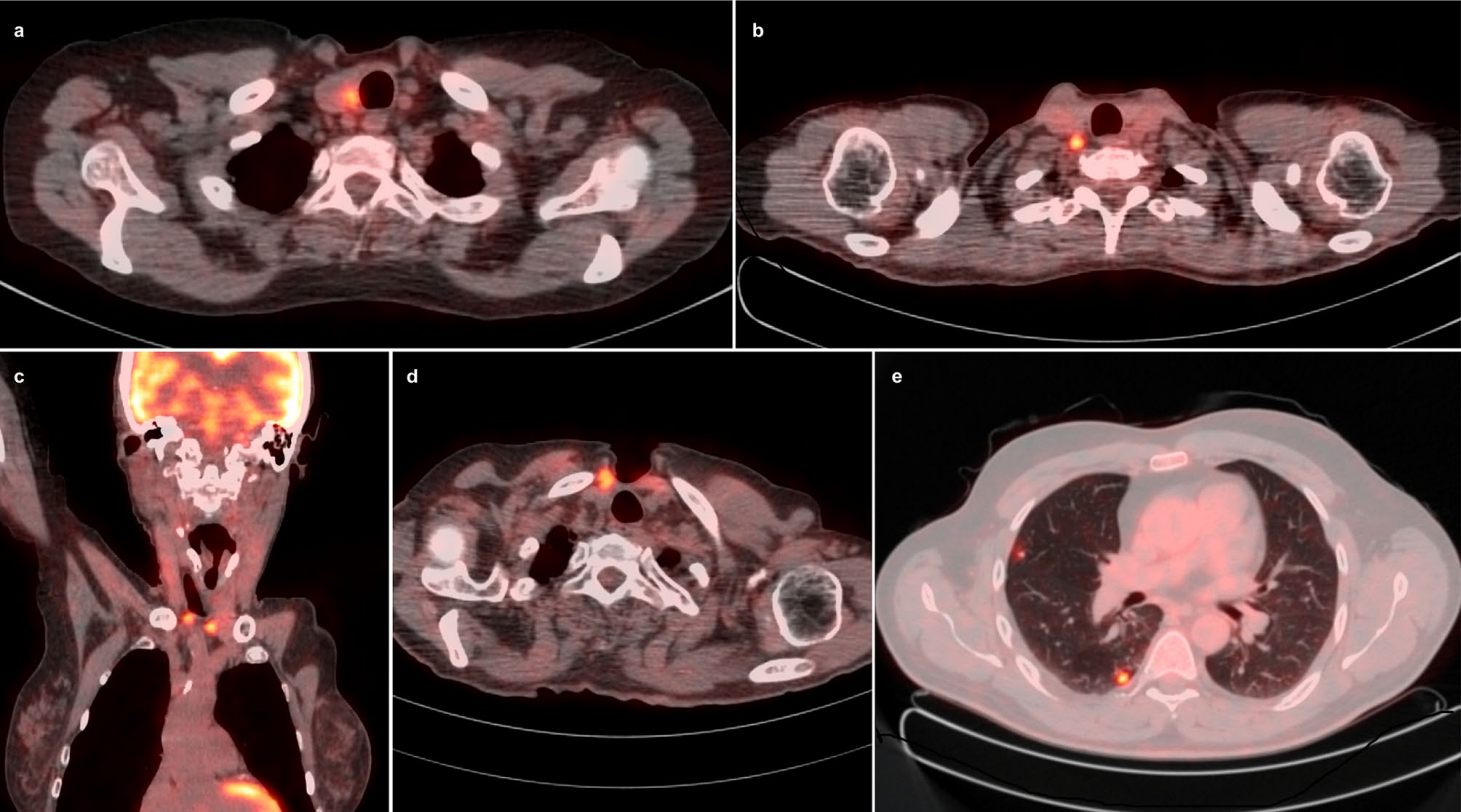
Examples of [^18^F]FDG PET/CT scans performed in patients with TENIS syndrome. (**a**) 70-year-old woman with papillary thyroid carcinoma with thyroglobulin level 17.28 ng/ml. PET scan detected an [^18^F]FDG-avid metastatic lesion in the thyroidectomy bed. (**b**) A 61-year-old woman with papillary thyroid carcinoma and thyroglobulin level 4.03 ng/ml. PET scan showed [^18^F]FDG-avid metastasis to the lymph node in the lateral neck compartment. (**c**, **d**) A 73-year-old woman with thyroid follicular carcinoma and thyroglobulin level 53.81 ng/ml. PET scan showed [^18^F]FDG-avid recurrent foci in subcutaneous tissue and muscles. (**e**) A 42-year-old man with thyroid follicular carcinoma and thyroglobulin level 202.60 ng/ml. PET scan detected [^18^F]FDG-avid lung metastases

In addition to the absolute level of Tg, the growth dynamics of this marker are important. Giovanella et al. indicated that the accuracy of [^18^F]FDG PET/CT increases significantly when the unstimulated Tg levels are > 5.5 ng/ml or when the Tg doubling time (TgDT) is < 1 year [77]. Conversely, Albano et al. identified TgDT ≤ 2.5 years as a predictive factor of positive [^18^F]FDG PET/CT results (sensitivity, 93%; specificity, 87%; AUC = 0.911) and demonstrated that it is a more effective criterion for selecting patients for PET imaging than absolute Tg level [79]. A recent meta-analysis confirmed the value of TgDT as a predictive factor for [^18^F]FDG PET results, as well as for response to treatment, disease recurrence, and overall survival (OS) in patients with DTC [80]. Examples of [^18^F]FDG PET/CT results in patients with TENIS syndrome are presented in Fig. 3.

The indication for [^18^F]FDG PET/CT in patients with TENIS syndrome should be determined after considering the clinical context. Firstly, low serum Tg levels do not always indicate a small tumour burden; in PDTC and certain aggressive histological subtypes of DTC, Tg expression can be decreased, resulting in lower production and secretion of Tg [81, 82]. Secondly, in TgAb-positive patients, the measurement of Tg levels is unreliable due to Tg assay interference [83]. In this situation, TgAb can serve as an imprecise surrogate tumour marker [84]. Total thyroidectomy followed by RAI treatment should eliminate the antigens required for TgAb production, leading to a gradual decrease in TgAb levels, with their disappearance typically occurring within a median of 3 year [85]. Thus, the presence of TgAb, and especially increasing serum TgAb levels, might indicate persistent and/or recurrent disease [86]. The diagnostic performance of [^18^F]FDG PET/CT for the detection of recurrence in DTC patients with negative [^131^I]I WBS and increased TgAb levels appears to be as high as in classic TENIS syndrome, as indicated by the meta-analysis by Bang et al. [51]. Another recent meta-analysis by Albano et al. reported pooled sensitivity and specificity of [^18^F]FDG PET/CT in this clinical scenario to be 84% and 82%, respectively [87]. Several studies proposed potential cut-off value of TgAb level with best accuracy to predict [^18^F]FDG PET/CT results, but their findings vary considerably and are difficult to compare due to the use of different TgAb assays [88, 89].

The impact of TSH (thyroid stimulating hormone) stimulation on the diagnostic value of [^18^F]FDG PET/CT in patients with DTC remains controversial. A meta-analysis by Ma et al. demonstrated that [^18^F]FDG PET performed under TSH stimulation identifies a greater number of patients with PET true-positive lesions, a higher number of PET-detected lesions, and a higher tumour-to-background ratio than [^18^F]FDG PET under TSH suppression. Moreover, PET scans performed under TSH stimulation alter the clinical management in 9% of patients [90]. However, more recent analyses involving a larger number of patients did not show statistically significant differences in the diagnostic accuracy of [^18^F]FDG PET/CT after stratifying patients according to TSH stimulation status [51, 91].

**Fig. 4:**
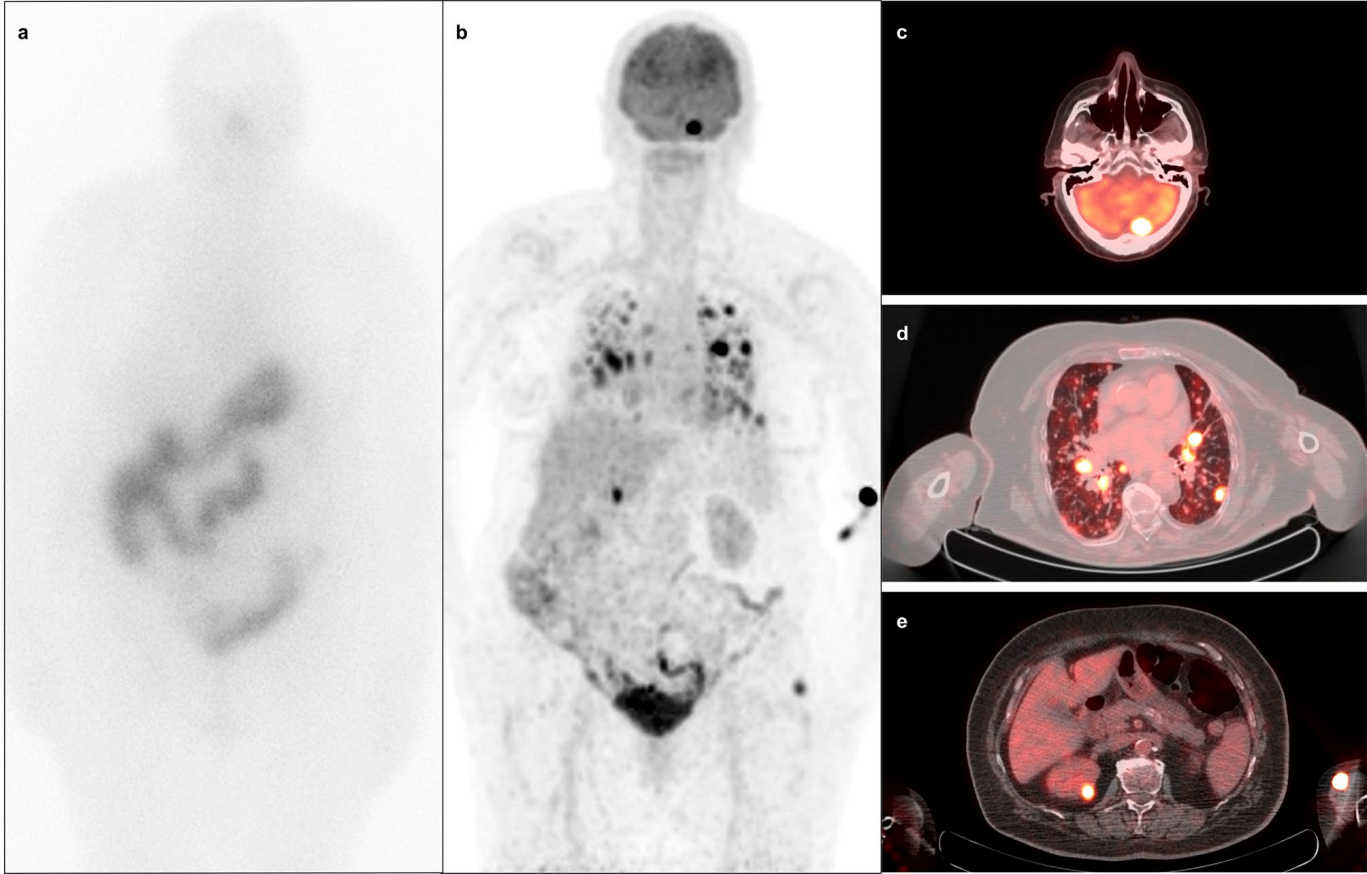
A 79-year-old female presented with high-risk papillary thyroid carcinoma after total thyroidectomy and central neck lymph node dissection, radiotherapy to the neck and upper mediastinum, and multiple cycles of [^131^I]I therapy with a total activity of 22.6 GBq. Post-therapeutic [^131^I]I WBS performed during the last radioiodine treatment was negative (**a**). The patient had an [^18^F]FDG PET/CT scan performed due to elevated thyroglobulin levels (202.3 ng/ml), which revealed [^18^F]FDG-avid metastatic lesions in the cerebellum, lungs, and right kidney (**b**-**e**)

Up to half of patients with PDTC and a significant proportion of patients with high-grade DTC become resistant to RAI during the follow-up period, which markedly limits the utility of [^131^I]I WBS and thereby increases the importance of [^18^F]FDG PET/CT in the management of these patients [6, 12]. Yang et al. assessed the utility of [^18^F]FDG PET/CT in high-risk DTC patients in various clinical settings; the greatest clinical benefits from [^18^F]FDG PET/CT were obtained in patients with lesions detected on [^131^I]I WBS that did not correspond to conventional imaging findings or were not proportional to stimulated Tg level, as well as in those with aggressive histological subtypes of DTC [5]. [^18^F]FDG PET/CT performed within 6 months after RAI therapy in patients with high-risk DTC and PDTC helps predict the treatment response, has a high impact on TNM staging and clinical management, and enables the early introduction of multimodal treatment [6, 92, 93]. An example of the use of [^18^F]FDG PET/CT in a patient with high-risk DTC during follow-up after initial treatment is shown in Fig. 4.

In addition, in some cases of OCA, conventional monitoring approaches including neck ultrasound, serum Tg measurement, and [^131^I]I WBS may be insufficient. Lopez-Penabad et al. reported that only 38% of patients with known OCA metastases show RAI uptake, and in patients with known lung or bone metastases, the uptake rate is < 10% [11]. Furthermore, patients with OCA may have distant metastases despite undetectable Tg levels and negative neck ultrasound results [94]. By contrast, [^18^F]FDG PET/CT demonstrates excellent diagnostic accuracy in patients with OCA, overperforming both [^131^I]I WBS and morphological imaging [95–97]. Plotkin et al. reported that [^18^F]FDG PET/CT detects OCA recurrence with a sensitivity of 92%, specificity of 80%, PPV of 92%, and NPV of 80% [97]. Many authors support the routine use of [^18^F]FDG PET/CT in both, the initial postoperative staging and the follow-up of OCA patients [95–97].

### Role of [^18^F]FDG PET/CT in novel targeted therapies for metastatic thyroid cancer

Understanding the signalling pathways involved in thyroid tumorigenesis has led to the development of novel molecular targeted therapies — tyrosine kinase inhibitors (TKIs) [98]. Multikinase inhibitors (MTKIs) have multiple targets associated with the pathogenesis of thyroid cancer (e.g., RET, KIT, BRAF, MET and EGFR) and neoangiogenesis processes (e.g., VEGFR and PDGFR). MTKIs include sorafenib, lenvatinib and cabozantinib, which are used in the treatment of advanced RAI-refractory DTC. Newer selective inhibitors target specific protein kinases such as RET kinase (selpercatinib and pralsetinib) and tropomyosin-related kinase (larotrectinib and entrectinib), and are used in tumours harbouring RET and neurotrophic tyrosine receptor kinase gene fusions. Additionally, combined inhibition of MEK and BRAF using dabrafenib and trametinib is an effective therapeutic option for BRAF V600E-mutated ATC. Evidence from the literature suggests that [^18^F]FDG PET/CT may serve as a valuable tool for patient qualification and evaluation of treatment response to these innovative drugs.

Marotta et al. [99] evaluated the role of [^18^F]FDG PET/CT performed prior to and 15 days after initiating sorafenib treatment in patients with advanced RAI-refractory DTC. Baseline mean SUVmax values in target lesions were significantly higher in patients who showed disease progression than in those who responded to treatment (*p* = 0.001). Early [^18^F]FDG PET/CT scans performed 15 days after the initiation of sorafenib showed that average SUVmax values decreased to a greater extent in patients who showed a clinical benefit from treatment than in non-responders (*p* = 0.002). However, there was no significant correlation between baseline and early [^18^F]FDG PET/CT results and progression-free survival (PFS). The authors concluded that baseline [^18^F]FDG PET/CT assessment may predict the radiological response to treatment, whereas early [^18^F]FDG PET/CT evaluation is effective for the rapid identification of non-responding patients, thereby preventing unnecessary therapy [99].

Another group of researchers analysed the role of [^18^F]FDG PET/CT in the evaluation of metabolic response and outcome prediction in patients with progressive RAI-refractory DTC treated with lenvatinib [100]. In most patients, the greatest metabolic response to lenvatinib was observed at the first [^18^F]FDG PET/CT scan, performed 4 weeks after the initiation of treatment; however, in some patients, this response was observed later during follow-up. The metabolic response detected by the [^18^F]FDG PET/CT scan performed after 4 weeks of lenvatinib was significantly correlated with the morphological response obtained in the CT performed after 8 weeks of treatment and later during follow-up. This enabled the early identification of patients who would not benefit from therapy. Moreover, patients with a positive metabolic response at the [^18^F]FDG PET/CT scan performed after 4 weeks of lenvatinib had a significantly longer median OS than non-responders (36.53 vs. 11.28 months) [100].

Additional studies have further validated the prognostic role of [^18^F]FDG PET in the early assessment of thyroid cancer patients treated with MTKIs [101–105]. Functional tumour response assessment by [^18^F]FDG PET outperforms morphological response assessment by CT because the therapeutic effect of TKIs is detected earlier by PET than by CT as a decline in PET-semiquantitative parameters [101, 102]. However, the optimal timing for [^18^F]FDG PET after the initiation of treatment remains controversial. Takeuchi et al. reported that the decrease in [^18^F]FDG uptake is already visible in PET scans performed 1 week after the initiation of lenvatinib therapy, thus enabling the prediction of treatment outcomes [101]. By contrast, the previously cited study by Valerio et al. suggests that a positive metabolic response may occur even after more than 4 weeks of treatment [100].

TKIs such as selumetinib, lenvatinib, trametinib, dabrafenib, vemurafenib and larotrectinib can restore the radioiodine uptake in RAI-refractory thyroid cancer patients [106–110]. Short-term (3–6 weeks) therapy with these TKIs restored or increased radioiodine uptake in 35–60% of patients, thus allowing subsequent RAI therapy [106–110]. In these studies, [^18^F]FDG PET/CT was used to determine the indication for redifferentiation therapy, for assessment of treatment response, and as follow-up [106, 107, 109, 111]. Weber et al. suggested that [^18^F]FDG PET/CT can serve as a predictive factor of the response to redifferentiation therapy [108]. These researchers evaluated the efficacy of redifferentiation therapy through genotype-guided MAPK inhibition: BRAF*V600E-*wild type patients were treated with trametinib, whereas patients with the BRAF*V600E* mutation received combination treatment with trametinib and dabrafenib. Redifferentiation was achieved in 7 of 20 (35%) patients. Subsequent RAI therapy led to a decrease in Tg levels in 57% (4/7) of the patients. The peak standardized uptake value (SUVpeak) of metastatic lesions on baseline [^18^F]FDG PET/CT significantly predicted the redifferentiation rate: redifferentiation was successful in 6 of 9 (66.7%) patients with a mean SUVpeak < 10 and unsuccessful in all 11 patients with a higher SUVpeak (*p* = 0.01) [108].

To date, there are no large-scale studies evaluating the role of [^18^F]FDG PET in patients with ATC treated with molecular targeted therapies. Only case reports and case series are available, suggesting that [^18^F]FDG PET/CT is effective for the qualification and assessment of treatment response with these drugs [112–116]. A case series of three patients with stage IVB and IVC ATC used short-term mutation-based “neoadjuvant” therapy [116]. One patient with a BRAF mutation received combination therapy with dabrafenib and trametinib, whereas the two BRAF-wild type patients were treated with a combination of pembrolizumab and lenvatinib for 4 weeks. Whole-body [^18^F]FDG PET/CT scans were performed at diagnosis and after 4 weeks of “neoadjuvant” treatment. Restaging PET showed significant tumour reduction and reduced glucose uptake in all three patients, who subsequently underwent surgical resection of the primary tumour; R0 resection status was achieved in two patients and R1 in one patient [116]. The efficacy of [^18^F]FDG PET for monitoring ATC patients treated with targeted therapies needs to be confirmed in larger patient cohorts.

### Prognostic value of [^18^F]FDG PET/CT

According to ATA guidelines, [^18^F]FDG PET/CT should be considered as a prognostic tool in thyroid cancer patients with metastatic disease to identify both lesions and patients at the highest risk of rapid progression and disease-specific mortality [2]. Uptake of [^18^F]FDG in metastatic lesions is a recognized negative prognostic factor for OS, whereas RAI uptake is prognostic for stable disease [64, 96, 117, 118]. In a multivariate analysis of 400 patients with follicular cell-derived thyroid carcinoma, only age > 45 years and [^18^F]FDG uptake in metastatic lesions were statistically significant predictive factors of OS, whereas initial stage, histology, serum Tg and RAI uptake were not. A significant inverse relationship was found between survival and both the glycolytic rate of the most active lesion (SUVmax) and the number of [^18^F]FDG-avid lesions [119]. In OCA, the 5-year OS is 92% in patients with SUVmax < 10 and 64% in patients with SUVmax > 10, and each increase in SUVmax of one unit is associated with a 6% increase in mortality (*p* < 0.001) [96]. Masson-Deshayes et al. found that PFS is significantly worse in patients with metastatic DTC with > 10 [^18^F]FDG-avid lesions and a standardized uptake value corrected for lean body mass (SUL) peak > 5 [120]. Other PET-derived semiquantitative parameters evaluated by various authors that could be useful for the dynamic risk stratification of patients with RAI-refractory DTC include metabolic tumour volume (MTV), total lesion glycolysis (TLG) and the location of PET-positive lesions [121–123].

Wijewardene et al. proposed a new scoring system, I-PET, which identifies patients who have or are likely to become refractory to RAI [124]. I-PET combines information from [^131^I]I WBS and [^18^F]FDG PET imaging, assigning the results to one of four categories: I‐PET [0]: Iodine −/FDG −, I‐PET [1]: Iodine +/FDG −, I‐PET [2]: Iodine +/FDG + and I‐PET [3]: Iodine −/FDG +. Patients with [^18^F]FDG-positive lesions (I‐PET [2] and I‐PET [3]) were further classified into groups A and B according to SUVmax ≤ 5 or > 5, respectively. After a median follow-up of 40 months, disease progression was observed in 22% of patients with I‐PET [0], 40% with I‐PET [1], 63% with I‐PET [2] and 74% of patients with I‐PET [3]. I‐PET [2B] and I‐PET [3B] were associated with progression rates of 88% and 78%, respectively. Patients classified as I‐PET [3B] had an 8-fold greater mortality (*p* = 0.003) and were 9.6-fold more likely to initiate multikinase inhibitor therapy (*p* = 0.001) than patients in other I‐PET groups [124].

The prognostic value of [^18^F]FDG PET/CT has also been demonstrated in the context of ATC. Poisson et al. identified the volume of [^18^F]FDG uptake (≥ 300 ml) and the intensity of [^18^F]FDG uptake (SUVmax ≥ 18) as significant prognostic factors for survival, whereas the number of involved organs did not have a prognostic role [14]. Similar conclusions were drawn by Kim et al., who showed that [^18^F]FDG PET/CT parameters such as SUVmax, MTV, and TLG were significantly associated with poor prognosis (*p* < 0.001, *p* = 0.002 and *p* < 0.001, respectively) [54].

### Limitations

This narrative review aims to provide an overview of the current knowledge on the role of [^18^F]FDG PET/CT in the management of follicular cell-derived thyroid carcinoma. The primary limitation of narrative reviews is the subjectivity in the selection of studies, their interpretation, and the synthesis of available evidence. Therefore, it should not be considered as clinical guidelines, and patient care and treatment should always be based on official recommendations from scientific societies, considering the individual clinical context. Nonetheless, the authors believe that this review offers a comprehensive understanding of the topic, highlights gaps in the available evidence, and serves as a starting point for future research.

## Conclusions

[^18^F]FDG PET/CT is a valuable tool in almost every step of thyroid cancer diagnosis and treatment, from the evaluation of indeterminate thyroid nodules, through the planning of surgical treatment and RAI therapy, to the assessment of patients with biochemical recurrence, and has a tangible impact on clinical management. Its importance increases with the degree of tumour dedifferentiation. In DTC, its application is limited to a small subset of patients in whom disease has eluded the standard approach. However, in poorly differentiated and undifferentiated forms, it represents one of the most effective diagnostic modalities. [^18^F]FDG PET/CT has recently acquired an increasingly important role in the assessment of response to treatment with molecular targeted therapies. Metabolic response, expressed as a decrease in semiquantitative PET parameters, is detectable earlier than structural response. This enables the early identification of patients who will not benefit from a therapy burdened with numerous adverse events. Finally, [^18^F]FDG PET/CT serves as a powerful prognostic tool capable of identifying both lesions and patients at high risk of rapid disease progression and disease-specific mortality, which can lead to the design of treatment and monitoring strategies tailored to the specific needs of individual patients.

## Data Availability

No datasets were generated or analysed during the current study.

## Abbreviations

AJCC/UICC: American Joint Committee on Cancer/Union for International Cancer Control
ATA: American Thyroid Association
ATC: Anaplastic thyroid carcinoma
CT: Computed tomography
DHGTC: Differentiated high-grade thyroid carcinoma
DTC: Differentiated thyroid carcinoma
ETA: The European Thyroid Association
[^18^F]FCH: [^18^F]fluorocholine
[^18^F]FDG: 2-[^18^F]fluoro-2-deoxy-D-glucose
FNAB: Fine needle aspiration biopsy
FTC: Follicular thyroid carcinoma
IEFVPTC: Invasive encapsulated follicular variant papillary thyroid carcinoma
[^131^I]I WBS: Iodine-131 whole-body scan
MRI: Magnetic resonance imaging
MTKI: Multitargeted tyrosine kinase inhibitors
NCCN: The National Comprehensive Cancer Network
NIS: Sodium/Iodide symporter
NPV: Negative predictive value
OCA: Oncocytic carcinoma of the thyroid
OS: Overall survival
PDTC: Poorly differentiated thyroid carcinoma
PET: Positron emission tomography
PET/CT: Positron emission tomography/computed tomography
PFS: Progression-free survival
PPV: Positive predictive value
PTC: Papillary thyroid carcinoma
RAI: Radioactive iodine
SUL: Standardized uptake value corrected for lean body mass
SUV: Standardized uptake value
TBR: Tumour-to-background ratio
[^99m^Tc]Tc-MIBI: [^99m^Tc]Tc-methoxyisobutylisonitrile
TENIS: Thyroglobulin elevated negative iodine scintigraphy
TgAb: Anti-thyroglobulin antibodies
TgDT: Thyroglobulin doubling time
TKI: Tyrosine kinase inhibitor
TNM: Tumour-Node-Metastasis
TSH: Thyroid stimulating hormone
WDTC: Well-differentiated thyroid carcinoma
